# Platelets from Asthmatic Individuals Show Less Reliance on Glycolysis

**DOI:** 10.1371/journal.pone.0132007

**Published:** 2015-07-06

**Authors:** Weiling Xu, Nayra Cardenes, Catherine Corey, Serpil C. Erzurum, Sruti Shiva

**Affiliations:** 1 Lerner Research Institute, Cleveland, Ohio, United States of America; 2 Vascular Medicine Institute, University of Pittsburgh School of Medicine, Pittsburgh, Pennsylvania, United States of America; 3 Lerner Research Institute, Respiratory Institute, Cleveland Clinic, Cleveland, Ohio, United States of America; 4 Vascular Medicine Institute, Dept of Pharmacology & Chemical Biology, Center for Metabolism and Mitochondrial Medicine, University of Pittsburgh School of Medicine, Pittsburgh, Pennsylvania, United States of America; University of Alabama at Birmingham, UNITED STATES

## Abstract

Asthma, a chronic inflammatory airway disease, is typified by high levels of T_H_2-cytokines and excessive generation of reactive nitrogen and oxygen species, which contribute to bronchial epithelial injury and airway remodeling. While immune function plays a major role in the pathogenesis of the disease, accumulating evidence suggests that altered cellular metabolism is a key determinant in the predisposition and disease progression of asthma. Further, several studies demonstrate altered mitochondrial function in asthmatic airways and suggest that these changes may be systemic. However, it is unknown whether systemic metabolic changes can be detected in circulating cells in asthmatic patients. Platelets are easily accessible blood cells that are known to propagate airway inflammation in asthma. Here we perform a bioenergetic screen of platelets from asthmatic and healthy individuals and demonstrate that asthmatic platelets show a decreased reliance on glycolytic processes and have increased tricarboxylic acid cycle activity. These data demonstrate a systemic alteration in asthma and are consistent with prior reports suggesting that oxidative phosphorylation is more efficient asthmatic individuals. The implications for this potential metabolic shift will be discussed in the context of increased oxidative stress and hypoxic adaptation of asthmatic patients. Further, these data suggest that platelets are potentially a good model for the monitoring of bioenergetic changes in asthma.

## Introduction

Asthma is defined as chronic airway inflammation characterized by increased T_H_2-cytokines, such as IL-4 and IL-13, and excessive generation of nitric oxide (NO) and reactive oxygen species (ROS) that ultimately results in bronchial epithelial injury and airway remodeling [1,2]. While a number of factors contribute to the pathogenesis of asthma, accumulating evidence suggests that altered cellular metabolism may play an important role. For example, a strong link has been established between asthma and metabolic syndrome [3–5] and significant metabolic changes have been observed in patients with asthma [6].

On a cellular level, mitochondrial function is central in regulating metabolism. Mitochondrial oxidative phosphorylation utilizes substrate to generate ATP more efficiently than glycolysis. Notably, both NO and T_H_2 cytokines have been demonstrated to regulate both oxidative phosphorylation and glycolysis [7–9]. Beyond ATP production, mitochondrial metabolism contributes to cellular homeostasis through the production of reactive oxygen species (ROS) which has been shown to be crucial in immune responses and pathologic inflammation [10,11,12]. Consistent with a role for the altered metabolism in asthma pathogenesis, changes in mitochondrial appearance and function have been identified in airway cells in the ovalbumin (OVA) allergen-murine experimental asthma model [13,14], and linked to asthmatic features such as airway hyper-responsiveness and mechanistically to T_H_2-driven inflammation [15]. Further, In humans, mitochondrial numbers and oxygen consumption rate in airway smooth muscle cells from asthmatic individuals are greater than cells from healthy controls, although the mechanisms have not been identified [16]. Interestingly, dietary studies suggest that asthmatic individuals may have systemic changes in cellular bioenergetics [6,17–20]. For example, Picado *et al* showed that individuals with mild asthma are metabolically more efficient as compared to healthy controls as measured by body mass index over the time period of careful regulation of dietary energy intake [6].

Despite the recognition that metabolism is potentially altered systemically in asthma, it is unknown whether a change in bioenergetic function can be detected in circulating cells of asthmatic patients. Platelets contain several fully functional mitochondria and are metabolically active with ATP production greater than muscle [21]. Resting platelets also use glycolysis as a source of energy [22], with much of the resultant pyruvate directed to lactate production. Recent studies have shown that glycolysis and oxidative phosphorylation are tightly linked in the platelet [23,24]. Further, activated platelets have been found in the bronchial lavage fluid of asthmatic patients [25,26] and associated with airway hyper-responsiveness[27]. Platelets are also known to contribute to airway inflammation and remodeling through the secretion of cytokines [28,29] and mitogens[30–32] and by their direct interaction and activation of eosinophils [29,33–35].

We have recently validated a method to measure mitochondrial function in circulating human platelets [36]. Here, we utilize this method to test the hypothesis that asthmatic individuals have systemic changes in cellular energy pathways that are detectable in circulating platelets. Our data reveal that platelets from asthmatic individuals rely less on glycolysis and have increased tricarboxylic acid (TCA) cycle enzymatic activity. The implications of these altered pathways on asthma pathogenesis as well as the potential use of platelets to monitor asthma pathogenesis clinically will be discussed.

## Materials and Methods

### Population

All studies were approved by the Cleveland Clinic Institutional Review Board (IRB#10–1049). All studies were performed in accordance with the principles outlined in the Declaration of Helsinki and written informed consent was obtained from all subjects. Asthma was verified based on positive methacholine challenge and/or reversible airway obstruction. Healthy controls lacked cardiopulmonary symptoms and had normal spirometry and negative methacholine challenge. Spirometry was performed with an automated spirometer.

### Human Platelet Isolation

Platelets were isolated by differential centrifugation from human venous blood collected in citrate containing tubes as previously described [36]. Briefly, whole blood was centrifuged (150× g, 10 min) in the presence of Prostaglandin I_2_ (PGI_2_)(1 μg/ml)(Sigma-Aldrich, St. Louis, MO) to obtain platelet rich plasma. Platelets were subsequently pelleted from the platelet rich plasma by centrifugation at 1500× g for 10 min. The platelet pellets were washed with Erythrocyte Lysis Buffer (Qiagen, Valencia, CA) and PGI_2_. The final samples were re-suspended in modified Tyrode’s buffer (20 mM Hepes, 128 mM NaCl, 12 mM bicarbonate, 0.4 mM NaH_2_PO_4_, 5 mM Glucose, 1 mM MgCl_2_, 2.8 mM KCl, pH 7.4) prior to study.

### Measurement of TCA cycle activity in Platelets

Aconitase activity was measured in isolated platelets (50 μg) lysed by three cycles of freeze/thaw by spectrophotometrically measuring the formation of NADPH at 340 nm in the presence of isocitrate/isocitrate dehydrogenase. Succinate dehydrogenase activity was measured by monitoring the rate of ubiquinol reduction coupled to the colorimetric dye dichlorophenolindophenol at 600nm. Citrate synthase activity was measured by monitoring the rate of conversion of acetyl CoA and oxaloacetate to citrate spectrophotometrically at 412nm by coupling CoA production with the colorimetric indicator dithionitrobenzoic acid.

### Western blot Analyses

Whole cell lysates prepared as previously described [37]. Rabbit anti-Aconitase polyclonal Ab (Santa Cruz Biotechnology), and mouse anti- Complex III-2 (Molecular Probes, Inc., Eugene, OR) and β-actin monoclonal Ab (Sigma-Aldrich, St. Louis, MO) were used in western analyses.

### Ultrastructural analyses

Samples were prepared as previously described [38]. Briefly, samples were fixed at 4°C for more than 1 h in 0.1 M sodium cacodylate buffer, pH 7.4, containing 2.5% glutaraldehyde and 4% formaldehyde. Samples were embedded in Eponate12 kit, polymerized at 70°C for 48 h, trimmed, sectioned at 70 − 90 nm, poststained in saturated uranyl acetate and lead citrate, and examined with a transmission electron microscope (Philips CM12).

### Measurement of Oxygen Consumption Rate (OCR) and Extracellular Acidification Rate (ECAR) in Platelets

OCR and ECAR were measured in isolated platelets using the Seahorse Extracellular Flux (XF24) Analyzer (Seahorse Bioscience Inc. North Billerica, MA) as previously described [36]. Isolated platelets were diluted in unbuffered Dulbecco’s Modified Eagle’s Media (DMEM; at 37°C) to 50 ×10^6^ cells/ml and 500 μl of sample loaded per well in standard XF24 plates. DMEM contained 25mM glucose, 2mM glutamine and 1mM pyruvate. Plates were centrifuged to create an unactivated cell monolayer. Once in the XF24, the cells were consecutively treated with oligomycin A (2.5 μM), FCCP (carbonyl cyanide-ρ-trifluoromethoxyphenylhydrazone) (0.7 μM), 2-deoxyglucose (2-DG)(100 μM) and Rotenone (2 μM). Three measurements of OCR and ECAR were made after addition of the agents and a mix step. Cell number was confirmed at the end of the experiment by crystal violet staining. All measurements were normalized to cell number.

Proton leak was quantified as the rate of respiration in the presence of oligomycin A minus the rate in the presence of rotenone. ATP-linked respiration was the difference of basal respiration and oligomycin A rate. Non-mitochondrial oxygen consumption is the rate in the presence of rotenone.

### ATP Content

Measurement of ATP levels in platelets was measured by using a luciferase-based luminescence assay kit (PerkinElmer, Waltham, MA) and measuring the linear rate of luminescence over 5 minutes as previously described [36]. ATP content was normalized to 10^6^ platelets.

### Statistical Analyses

Data are shown as mean ± SE. All statistical comparisons are performed using the Student’s t-test, paired t-test or one-way ANOVA as appropriate. The level of significance for *P* was chosen at 0.05. All data were analyzed with statistical program JMP Pro 10 (SAS Institute, Cary, NC).

## Results

### Platelets from subjects with asthma show no change in mitochondrial number or morphology

**Fig 1:**
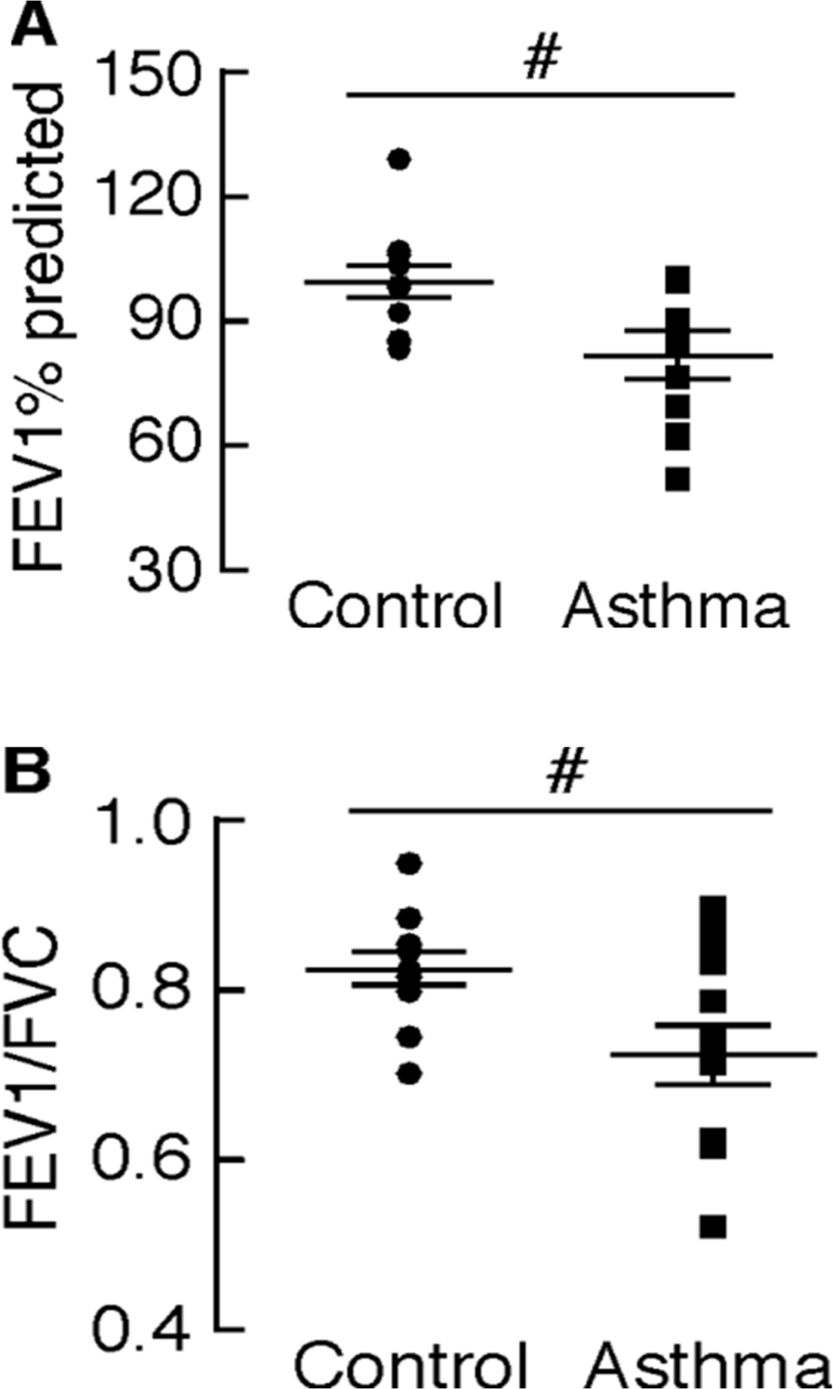
Asthmatics show airflow obstruction compared to healthy controls. Airflow obstruction as measured by (A) FEV1% predicted and (B) FEV1/FVC in asthmatic individuals compared with healthy control subjects. #p<0.05.

To investigate platelet metabolism in asthma, a study population including 13 healthy controls and 12 individuals with asthma was utilized (**Table 1**). The asthmatics had airway reactivity to methacholine, but all asthmatic subjects were non-severe, as defined by criteria of the American Thoracic Society [39]. This was demonstrated by assessment of airflow by measurement of forced expiratory volume in 1 second (FEV1)% predicted and the ratio of FEV1 to forced vital capacity (FVC) (**Fig 1**). These data demonstrate that although asthmatics had significantly lower airflow compared to control subjects, the airflow limitation was generally mild to moderate [FEV1% predicted: 99 ± 3 (controls) vs 81 ± 5 (asthma), P = 0.01; FEV1/FVC: 0.82 ± 0.02 (controls) vs 0.73 ± 0.03 (asthma), P = 0.03]. Asthmatics were well controlled for at least the prior 6 weeks or longer, and withheld medications prior to day of testing. The number of subjects assessed for each experiment is provided with each result.

**Table 1:** Features of Study Participants.

Characteristics	Controls (n = 13)	Asthma (n = 12)	*P* *
Mean age, yr	39 ± 3	35 ± 3	0.4
Gender, M/F	8/5	6/6	0.5
Ethnicity, C/AA/other	10/2/1	6/5/1	0.3
Heart rate (beats/min)	69 ± 5	70 ± 3	0.8
FEV_1_% predicted	99 ± 3	81 ± 5	0.01
FEV_1_/FVC	0.82 ± 0.02	0.73 ± 0.03	0.03
% Atopy	45	100	0.004

Definition of abbreviations: M, male; F, female; C, Caucasian; AA, African Amirican; FEV_1_, Forced expiratory volume in 1 second; FVC, Forced vital capacity;

* *P* value, asthma *vs*. controls.

**Fig 2:**
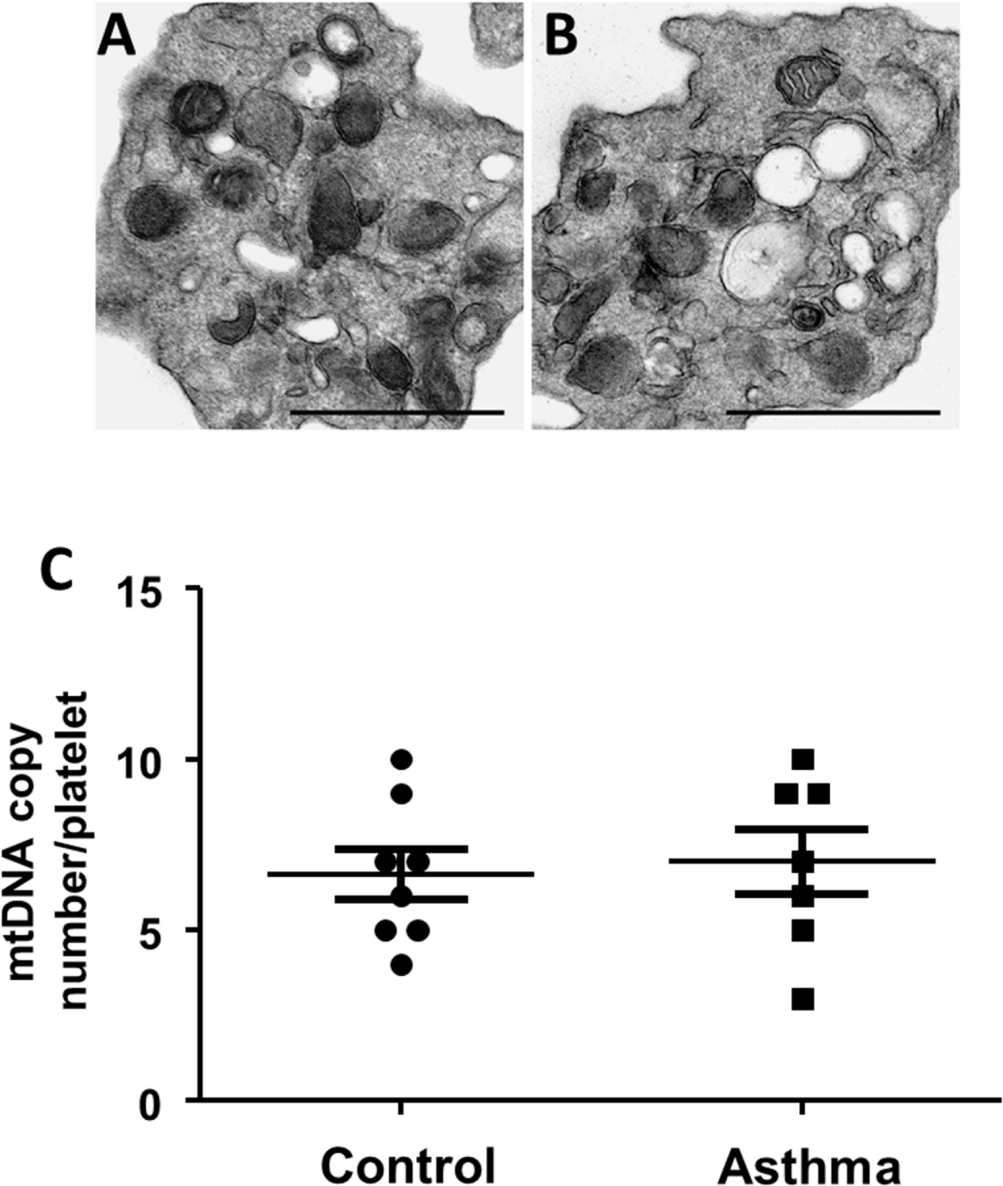
Platelets show no change in mitochondrial number and morphology in asthma. (A-B) Representative electron micrograph of platelet from (A) healthy control and (B) asthma (scale bars: 1 μm). (C) Mitochondrial DNA copy number per platelet in platelets from healthy and asthmatic individuals. n = 7 per group.

Electron microscopy of platelets from healthy and asthmatic individuals showed no overt differences in mitochondrial number or morphology (**Fig 2A and 2B**). Quantification of the number of mitochondria in each platelet demonstrated that there was no difference between the two groups (2.85 ± 0.40 vs 2.59 ± 0.38; asthma vs control; n = 10 platelets each from n = 4 subjects in each group). This was confirmed by measurement of mitochondrial DNA content in 7 different subjects in each group, which showed no significant changes in mitochondrial DNA copy number between groups (**Fig 2C**).

### Platelets show less reliance on glycolysis in asthma

**Fig 3:**
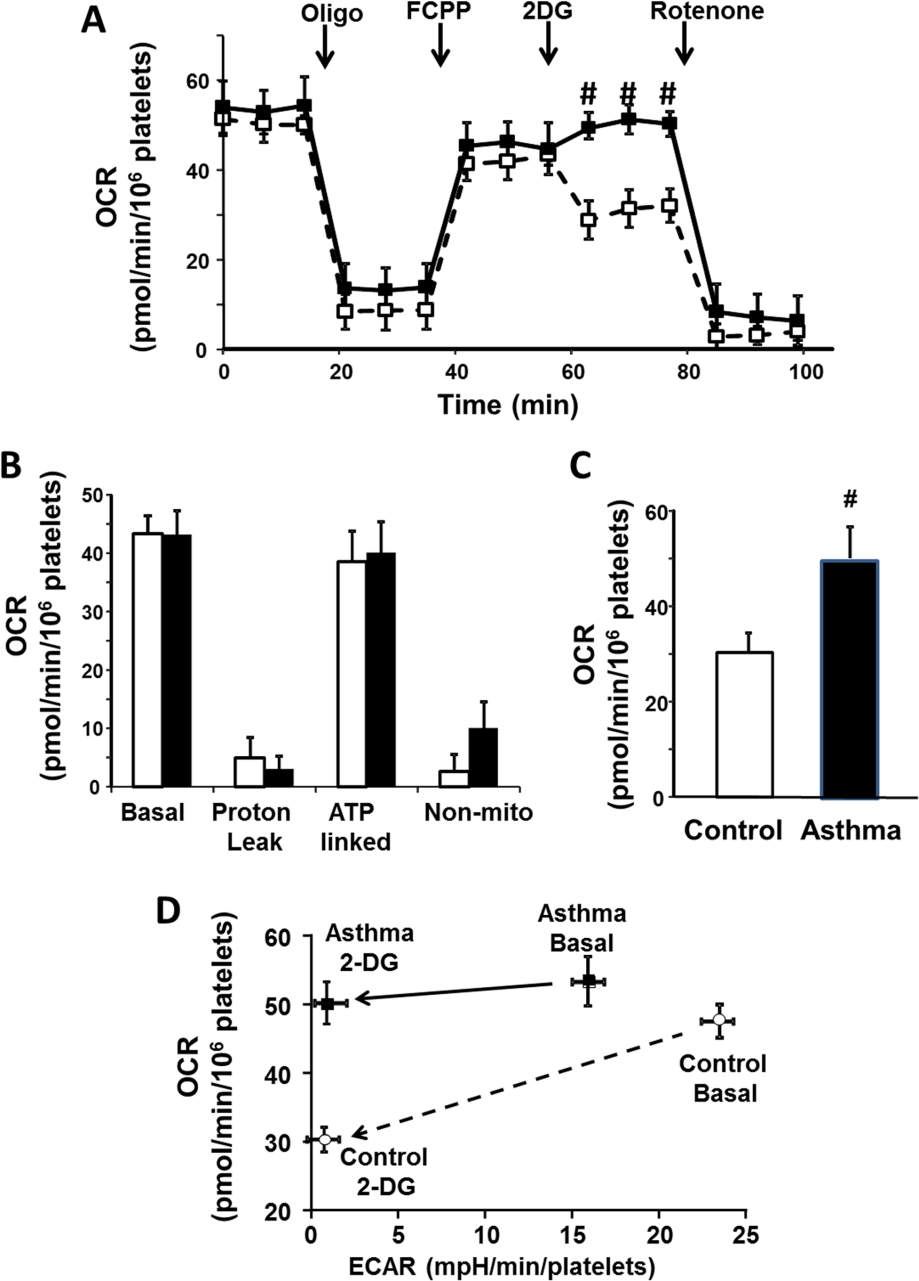
Platelets show less reliance on glycolysis in asthma. (A) Oxygen consumption trace for healthy (open squares) and asthmatic (filled squares). Basal rate is shown and arrows denote the addition of oligomycin A (oligo), FCCP, 2-deoxyglucose (2DG) and rotenone. (B) Quantification of basal rate, proton leak, ATP-linked respiration and non-mitochondrial oxygen consumption in platelets from healthy (open bars) and asthmatic (filled bars) subjects (calculated from traces such as those shown in panel A). (C) Quantification of oxygen consumption rate after the addition of 2-DG in healthy (open bars) and asthmatic (filled bars) platelets. (D) Changes in OCR as a function of ECAR in healthy (control; open circles) and asthmatic (Asthma; filled squares) platelets basally and after the addition of 2-DG. Arrows depict the shift after addition of 2-DG. Data are means ± SEM. #p<0.05. n = 12 for asthma, n = 13 for control.

To determine whether bioenergetics were altered in platelets isolated from patients with asthma, oxygen consumption rate was first measured in platelets isolated from both groups. Basal oxygen consumption rate (OCR) of platelets was similar among groups [OCR pmol O_2_/min/10^6^ platelets, control (n = 13) 47.8 ± 2.7, asthma (n = 12) 53.3 ± 6.0, *P* = 0.4] and respiratory rate was significantly decreased by the ATP synthase inhibitor oligomycin to a similar extent in both groups (**Fig 3A**). Treatment with rotenone, a pharmacological inhibitor of mitochondrial Complex I, affirmed that the OCR measured was predominantly mitochondrial with rotenone inhibiting 92±7% and 99.8±4% of oxygen consumption in platelets from asthma and control subjects respectively (**Fig 3A and 3B**). Analysis of this data demonstrates that there was no significant difference in ATP-linked respiration or proton leak between the two groups (**Fig 3B**). Notably, in the presence of deoxyglucose (2-DG), a competitive glucose analogue that inhibits glycolysis, oxygen consumption did not decrease in asthma, so that asthmatics had greater OCR than controls in the absence of glycolysis (**Fig 3A and 3C**). **Fig 3D** demonstrates this relationship between inhibition of glycolysis and the resulting drop in OCR observed in controls but not asthmatics. These data suggest that while asthmatic platelets are able to compensate for the inhibition of glycolysis-based ATP production, control platelets are more reliant on glycolysis, potentially due to substrate limitation.

**Fig 4:**
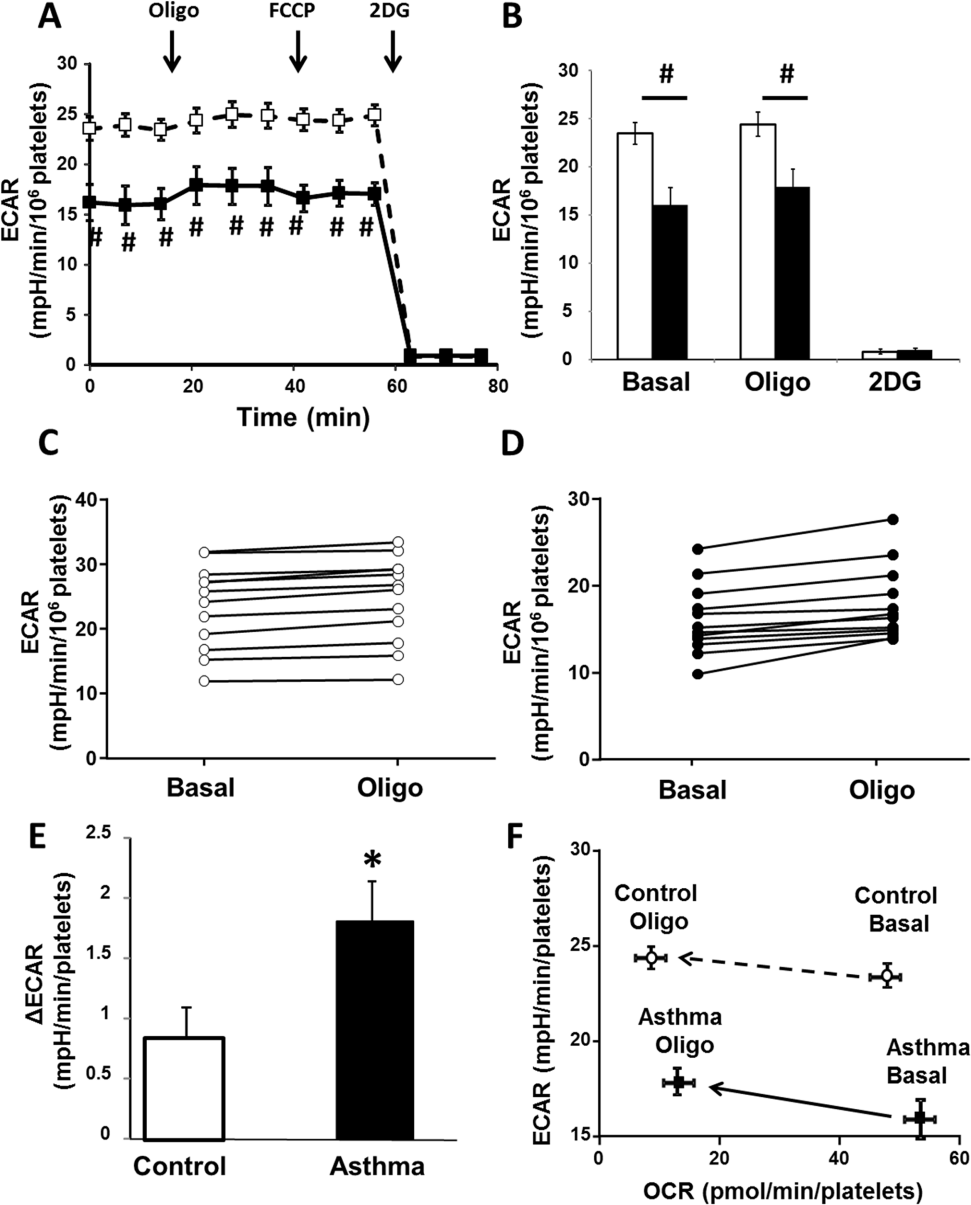
Platelets show decreased glycolytic rate in asthma. (A) Extracellular acidification rate (ECAR) trace in asthmatic (filled squares) and healthy controls (open squares) basally, after the addition of oligomycin A, FCCP and 2-DG. (B) Quantification of ECAR basally, after oligomycin A addition and 2DG treatment in healthy controls (open bars) and asthmatic platelets (filled bars). (C-D) ECAR basally and after oligomycin A addition for platelets from each (C) healthy control subject and (D) asthmatic individuals. (E) Quantification of the difference in ECAR from basal to oligomycin A addition in healthy (control)(white bar) and asthmatic individuals (black bar). (F) The change in ECAR as a function of OCR in healthy (open circles) and asthmatic (filled squares) platelets basally and after the addition of oligomycin A. Arrows show the shift in ECAR/OCR after oligomycin A addition. n = 12 asthma, n = 13 controls; *p<0.01; #p<0.05.

To determine whether glycolytic rate differed in asthma, we next compared basal glycolysis in platelets from asthma and healthy controls (**Fig 4A and 4B**). Evaluation of glycolysis was based on the measurement of extracellular acidification rate (ECAR), which increases due to glycolysis-dependent cellular proton production and export, a process that correlates with lactic acid formation. Basal ECAR in platelets from asthmatics (16.0±1.2 mpH/min/10^6^ platelets) was significantly lower than control platelets (23.5±1.9 mpH/min/10^6^ platelets)(**Fig 4A and 4B**). ECAR in both groups was inhibited ~99% by 2-DG as expected. Further, when ATP production by oxidative phosphorylation was inhibited using oligomycin A, there was a small but statistically significant increase in glycolytic rate (compared to basal rate) in each subject of both the healthy control (**Fig 4C**; P = 0.005) and asthmatic (**Fig 4D**; P<0.001) groups. Notably, this oligomycin A-induced increase (calculated by subtracting basal rate from oligomycin rate for each subject) was significantly greater in platelets from asthmatic than control individuals (**Fig 4E**). Thus, the relationship of ECAR/OCR is shifted in asthmatics compared to healthy controls in the presence of oligomycin (**Fig 4F**). These data suggest that platelets from asthmatic individuals show less reliance on glycolysis (and are more oxidative) than those from control subjects.

**Fig 5:**
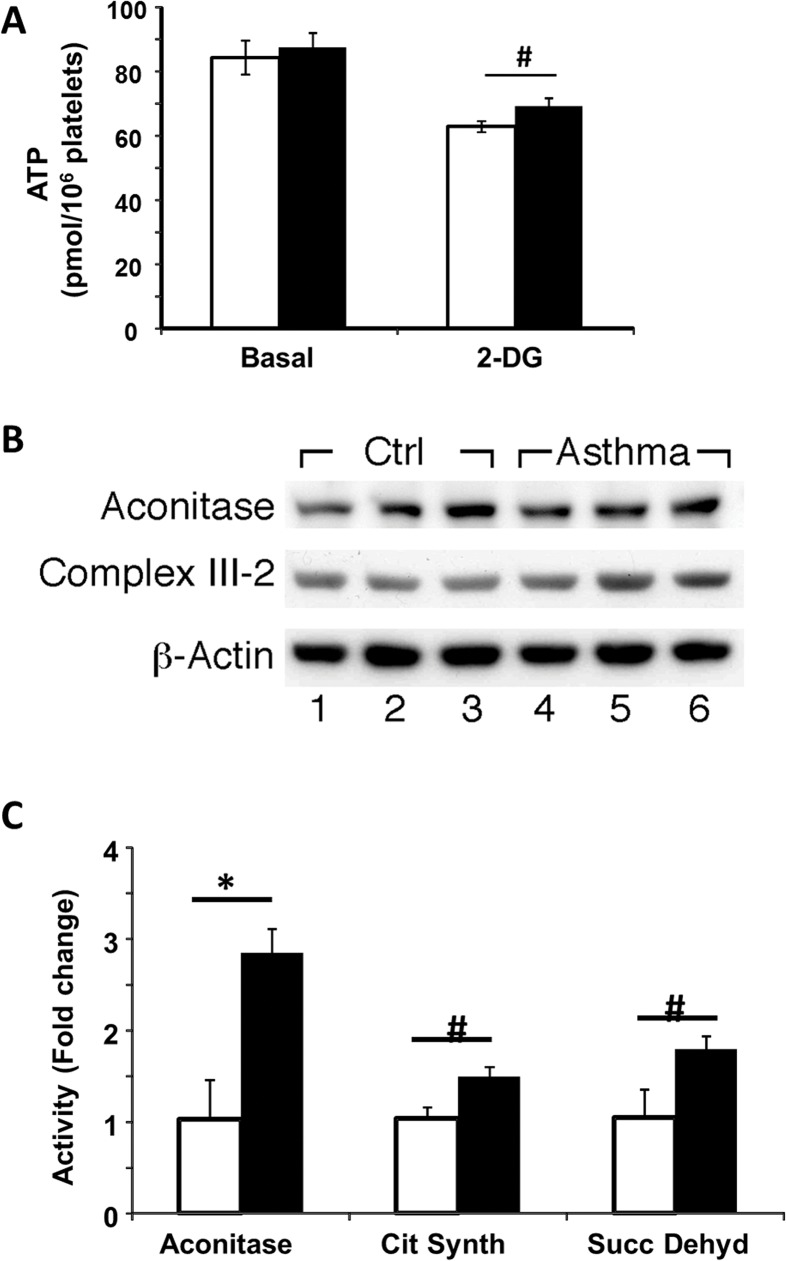
Greater TCA Cycle Activity in Asthma. (A) ATP content in control (white bars) and asthmatic (black bars) individuals basally and after inhibition of glycolysis by 2-DG. (B) Western analyses of aconitase expression in platelets. Asthmatic individials (lanes 4−6) had similar aconitase protein expression in platelets to healthy controls (lanes 1−3) by western blot. (C) Activity of aconitase, succinate dehydrogenase and citrate synthase in platelets from asthmatics (black bars) as a fold change of the activity of control platelets (white bars). n = 13 controls, n = 10 asthmatics. *p<0.01; #p<0.05.

ATP content in platelets from asthma (87.43 ± 4.61 pmol/10^6^ platelets; n = 7) was not significantly different from controls (84.37 ± 5.32 pmol/10^6^ platelets; n = 8; P = 0.8). However, ATP content in the presence of glycolytic inhibition was greater in platelets from asthmatics (69.12 ± 2.51 pmol/10^6^ platelets; n = 7) than controls (62.71 ± 1.54 pmol/10^6^ platelets, *P* = 0.04). These data indicate less glycolytic-reliance for cellular respiration in asthma (**Fig 5A**).

### TCA Cycle enzyme activity is increased in Asthma

The bioenergetics data presented above suggests that lactic acid production was decreased in asthma. This is potentially due to greater conversion of pyruvate in asthma to form oxaloacetate, which enters the TCA cycle to fuel oxidative phosphorylation. Thus, we next assessed the activity of the TCA cycle enzymes. Measurement of three TCA cycle enzymes, citrate synthase, aconitase and succinate dehydrogenase, demonstrated that while protein expression was unchanged, platelets from asthmatics showed significantly increased enzymatic activity (**Fig 5B and 5C**). These data support the concept of increased TCA cycle enzymatic activity in platelets from asthmatic individuals, fueling oxidative phosphorylation.

## Discussion

All cells have metabolic flexibility and the capacity to shift reliance to glycolysis relative to cellular respiration through molecular mechanisms that enable cells to adapt as needed for changes in nutrient substrates and/or energy demands. Our studies demonstrate that platelets of asthmatics had increased TCA cycle enzymatic activity and less dependence on glycolysis and on glucose for cellular respiration, which results in mitochondria with greater capacity for the aerobic production of ATP.

In this study, we provide evidence that supports a potential shift of cellular energy metabolism from glycolysis to mitochondrial respiration in asthma. There is substantial evidence that metabolic shifts, in particular to glycolytic pathways, have pathological consequences [38,40–42]. However, the shift from glycolysis to mitochondrial pathways may also be detrimental given that the electron transport chain is a significant source of reactive oxygen species. Thus, the redirection of metabolism toward mitochondrial metabolism could augment oxidative injury in asthma. Although mitochondria have been linked primarily to adverse consequences of greater ROS formation in asthma, some studies do suggest greater mitochondrial efficiency in energy production [6]. For example, individuals with mild asthma are metabolically more efficient as compared to healthy controls, *i*.*e*. body mass index is greater in asthma even with careful regulation of dietary intake equivalent to healthy individuals [6]. The strong consistent relationship between asthma and obesity [3] also suggests that asthma is associated with metabolic disease. Likewise, obesity has been identified as a major risk factor for the development of asthma [3–5]. Here we found similar extracellular acidification rate (ECAR) as controls but maintenance of oxygen consumption rate (OCR) and ATP production with glycolytic inhibition in asthmatic platelets. These data support a metabolic flexibility in asthmatic platelets that allows for ATP production with less reliance on glycolysis.

While the current study focuses on the role of glycolysis in asthmatic platelets, it is important to note that fuel sources other than glucose should be considered. The non-essential amino acid glutamine can be converted to glutamate by glutaminase and shuttled into the TCA cycle [43]. As glutamine (2mM) was present in the experimental conditions of the current study, a switch to glutaminolysis in the in the asthmatic platelets cannot be ruled out as a potential mechanism for the maintenance of oxygen consumption in the presence of glycolytic inhibition. Notably, while no previous reports exist of increased glutaminolysis in asthma, studies have documented increased plasma glutamine levels and altered glutaminase activity in airway epithelial cells of asthmatic patients [44,45]. Further study, modulating substrate availability, is warranted to determine whether glutamine is a major fuel source in asthmatic platelets.

These data provide new insights into metabolic origins of asthma. The ability to generate ATP despite depletion of substrate has conceivable benefits for adaptation and acclimatization for survival under conditions of food scarcity. Similarly, the ability to extract more oxygen for ATP production has clear benefit under conditions of hypoxia [46,47]. Life is limited by availability of oxygen in conditions of critical illness, during extreme exercise of elite athletes, and in challenging environments with limited inspired oxygen content. The findings in this study suggest that asthmatic individuals would thrive better under conditions of limited oxygen. In fact, asthmatic patients do very well with long-term residence at high altitude and have symptomatic improvement of asthma [48–50]. Intriguingly, the prevalence of asthma among elite athletes is much higher than asthma in the general population, suggesting that the condition of asthma potentially provides an advantage for aerobic extreme sports [51,52].

Notably, accumulating evidence suggests that circulating platelets play an integral role in the modulation of airway inflammation [25,28,33,34,53,54]. Platelets express surface IgE receptors[55,56], secrete a number of leukocyte chemotactic mediators[28,29,33,34,53], and secrete mitogens involved in tissue remodeling [30–32]. Additionally, platelet activation is increased in asthmatic patients[53,57], circulating platelet aggregates have been identified in humans after an asthma attack[57,58], bronchoalveolar lavage fluid from asthmatic subjects contain activated platelet products[25,26] and platelet activation has been associated with bronchial hyper-responsiveness[27]. Perhaps most importantly, platelets can directly interact with eosinophils, forming circulating complexes that facilitate intercellular signaling, resulting in the priming and activation of eosinophils [29,33–35]. Complex formation induces the surface expression of integrins on the eosinophil, priming it for recruitment and adhesion to the inflamed lung [29,59]. A number of studies now link altered platelet metabolism to augmented platelet activation and function [36,60,61]. Thus, while more research is required, it is interesting to speculate that platelet metabolism may represent a therapeutic target to decrease the severity of airway inflammation.

Finally, these data confirm that beyond local airway effects, asthmatic individuals also have systemic changes in cellular energetics. Notably, these data indicate a capacity for greater oxygen utilization and more efficient energy production from substrate in platelets, similar to what has previously been reported in the airways. These data support the idea that with further study, platelet bioenergetics could potentially be utilized as biomarker to monitor the severity of asthma or bioenergetic alterations that occur with therapy.
